# Getting the right grasp on executive function

**DOI:** 10.3389/fpsyg.2014.00285

**Published:** 2014-04-07

**Authors:** Claudia L. R. Gonzalez, Kelly J. Mills, Inge Genee, Fangfang Li, Noella Piquette, Nicole Rosen, Robbin Gibb

**Affiliations:** ^1^Department of Kinesiology, The Brain in Action Laboratory, University of LethbridgeLethbridge, AB, Canada; ^2^Department of Modern Languages, University of LethbridgeLethbridge, AB, Canada; ^3^Department of Psychology, Univeristy of LethbridgeLethbridge, AB, Canada; ^4^Department of Education, University of LethbridgeLethbridge, AB, Canada; ^5^Department of Neuroscience, University of LethbridgeLethbridge, AB, Canada

**Keywords:** grasping movements, left hemisphere, space use, development, frontal lobe, handedness, assessment, intervention

## Abstract

Executive Function (EF) refers to important socio-emotional and cognitive skills that are known to be highly correlated with both academic and life success. EF is a blanket term that is considered to include self-regulation, working memory, and planning. Recent studies have shown a relationship between EF and motor control. The emergence of motor control coincides with that of EF, hence understanding the relationship between these two domains could have significant implications for early detection and remediation of later EF deficits. The purpose of the current study was to investigate this relationship in young children. This study incorporated the Behavioral Rating Inventory of Executive Function (BRIEF) and two motor assessments with a focus on precision grasping to test this hypothesis. The BRIEF is comprised of two indices of EF: (1) the Behavioral Regulation Index (BRI) containing three subscales: Inhibit, Shift, and Emotional Control; (2) the Metacognition Index (MI) containing five subscales: Initiate, Working Memory, Plan/Organize, Organization of Materials, and Monitor. A global executive composite (GEC) is derived from the two indices. In this study, right-handed children aged 5–6 and 9–10 were asked to: grasp-to-construct (Lego® models); and grasp-to-place (wooden blocks), while their parents completed the BRIEF questionnaire. Analysis of results indicated significant correlations between the strength of right hand preference for grasping and numerous elements of the BRIEF including the BRI, MI, and GEC. Specifically, the more the right hand was used for grasping the better the EF ratings. In addition, patterns of space-use correlated with the GEC in several subscales of the BRIEF. Finally and remarkably, the results also showed a reciprocal relationship between hand and space use for grasping and EF. These findings are discussed with respect to: (1) the developmental overlap of motor and executive functions; (2) detection of EF deficits through tasks that measure lateralization of hand and space use; and (3) the possibility of using motor interventions to remediate EF deficits.

## Introduction

Historically, neuropsychological evidence has highlighted the role of the frontal cortex in the planning and execution of behavior (Kolb and Whishaw, 2009). Patients with frontal lobe injury present with a host of motor and cognitive disturbances. In the motor domain, frontal lobe injury could lead to deficits in gross motor function (e.g., impaired posture and gait) and/or fine motor control (e.g., impaired reaching and grasping). In the cognitive domain some of the most commonly disrupted functions include: initiation, planning, purposive action, self-monitoring, self-regulation, and volition (Stuss, 2011). This has led to the understanding that the frontal lobe is the area that supports executive function (EF). EF is a blanket term that is considered to include attentional control, self-regulation, inhibition, working memory, goal setting, planning, problem solving, mental flexibility, and abstract reasoning (Diamond and Lee, 2011).

Early in life, children learn and refine a host of motor skills that will have a phenomenal impact on later cognitive function. In fact, there is evidence that the time scales for development of these functions imbricate (see Diamond, 2000; for a review, Diamond, 2007). In addition, imaging studies have shown overlapping activation of motor function and EF in the frontal lobe, in particular the dorsal premotor cortex, which responds to planning, selection, organization, and execution of actions (Abe and Hanakawa, 2009; Hanakawa, 2011). In a retrospective study Piek et al. (2008) correlated data gathered in the preschool years using the Ages and Stages Questionnaire (ASQ) for gross motor trajectory with later performance on the Wechsler Intelligence Scale in elementary school. They found a high correlation between the two, once socioeconomic status was controlled for. Furthermore, they showed a predictive relationship between motor outcomes and working memory function. They and others have concluded that abnormalities in motor performance may be an important basis for the detection of later cognitive impairments (Piek et al., 2008; Butcher et al., 2009; Iverson, 2010). In fact, Kirby et al. (2008) report that more than 50% of university and college students with motor difficulties also suffer from difficulties with executive function. This evidence highlights the enduring nature of the relationship between motor and executive function.

An emerging research field is providing evidence of the interrelatedness of motor and executive functions, particularly in the planning domain (Pennequin et al., 2010; Thibaut and Toussaint, 2010; van Swieten et al., 2010; Jongbloed-Pereboom et al., 2013; and see Rosenbaum et al., 2012 for a review). For example, recently Jongbloed-Pereboom et al. (2013) asked 3–10 years old children to grasp a wooden sword and place it into a fitted aperture. The handle of the sword was placed in one of six different orientations. The authors documented the grip type that participants used and analyzed it with respect to end-state comfort. It was found that action planning increased from 3 to 10 years of age. Ten year olds behaved more like adults such that they preferred an awkward initial grasp to assure a final end-state comfort. Authors conclude that a cognitive component directly related to anticipatory planning subserves the performance of this task. Given that both planning and inhibition are critical components of EF, this evidence suggests a rich connection between cognition and action. Based on this literature, we hypothesized that measures of motor performance and EF could be mutually predictive. A motor action that we perform hundreds of times each day is reaching and grasping. Grasping has been shown to develop as early as 6 months of age and can be reliably assessed by age one (Michel et al., 2006; Jacquet et al., 2012; Sacrey et al., 2012, 2013). Using such an ecologically-valid measure of motor performance we sought to investigate its possible relationship with EF. If this relationship is established, the implications are paramount for improving life-long success, for three reasons. First, skilled motor ability can be readily assessed earlier than EF. Second, EF has been shown to be a better predictor of school success than IQ (Blair and Razza, 2007; Diamond and Lee, 2011; Masten et al., 2012). Third, if developmental delays are detected, interventions for both motor skill and EF training can be implemented immediately to prevent academic setbacks later in life.

In the present investigation we examined EF and motor performance in two groups of children; 5–6 and 9–10 year olds. We used the Behavioral Rating Inventory of Executive Function (BRIEF; Gioia et al., 2000) to assess EF and two reaching and grasping tasks to assess motor performance. The BRIEF was developed as an ecologically valid model to assess children's executive functions (Gioia et al., 2000). According to Gioia and Isquith (2004), the BRIEF was designed as “a means of culling and standardizing the rich information provided by parents and teachers in a more reliable and efficient manner with known psychometric properties.” This test has been widely used to assess executive function in normal and clinical populations and there have been several validity studies demonstrating its effectiveness (for review see Donders, 2002; Strauss, 2006). Moreover, a recent study corroborated the effectiveness of the BRIEF as a tool to assess EF, as it was found that BRIEF measures correlated with in-lab behavioral measures (Lalonde et al., 2013). Furthermore, studies have shown strong correlations with academic performance and scores obtained with the BRIEF (e.g., Waber et al., 2006).

Reaching and grasping was assessed using two well-studied grasping tasks: grasp-to-place and grasp-to-construct (Gonzalez et al., 2006, 2007; Gonzalez and Goodale, 2009; Gallivan et al., 2011; Sacrey et al., 2013; Stone et al., 2013; Stone and Gonzalez, 2014). In the grasp-to-place task participants are asked to reach for and grasp wooden blocks with colors or numbers and place them into a box. The grasp-to-construct task requires individuals to locate, reach for and grasp plastic blocks (LEGO®) of different size, shape, and color in order to replicate a model based on a sample. Because the grasp-to-construct task demands that participants plan and strategize in order to reproduce the sample as fast and accurately as possible, we hypothesized that this task, in particular, would be sensitive to a relationship between motor and executive function.

## Materials and methods

### Participants

A total of 40 children took part in the study. All children were identified as right-handed according to a modified version of the Edinburgh Handedness questionnaire (Oldfield, 1971; completed by each parent; see Stone et al., 2013 for full version of the questionnaire). Thirty-one children had previously participated in a psychological study at the University of Lethbridge (U of L), at which time their parents had opted to receive e-mail notifications of future studies at the U of L. The remaining children were recruited through either acquaintances of the authors, or at a booth during a public children's festival. Nineteen individuals comprised the “younger” age group of 5 and 6 year olds (11 females; M ± *SD* age = 5.98 ± 0.53 years) and 21 individuals comprised the “older” age group of 9 and 10 year olds (10 females; M ± *SD* age = 9.88 ± 0.51 years). Participants were healthy, with no evidence of neurological impairment. Participants were naïve to the purpose of the study and informed parental consent, as well as child verbal consent, was obtained prior to participation.

### Procedure

#### Parent questionnaires

After informed consent was obtained, the parent accompanying the child participant was given three paper-based questionnaires to be completed: (1) a participant information sheet that consisted of general questions regarding the child's motor, cognitive, and language development. (2) a modified version of the Edinburgh Handedness Inventory (to be filled out with the child's hand preferences in mind); and (3) the BRIEF (Gioia et al., 2000). For the BRIEF, the parent was asked to rate 86 everyday behaviors over the past 6 months as never occurring, sometimes occurring, or often a problem for their child. Each behavior belongs to one of eight subscales that represent unique facets of executive function (Gioia and Isquith, 2004): (1) Inhibit (resist or delay an impulse); (2) Shift (change problem-solving strategies); (3) Emotional Control (appropriately modulate affective reactivity); (4) Initiate (begin a task or activity, generate ideas); (5) Working Memory (hold information in mind for the purpose of completing a task); (6) Plan/Organize (anticipate events, set goals, and develop steps to carry out a task); (7) Organization of Materials (establishing and maintaining order to systematically carry out a task); (8) Monitor (check action to assure appropriate attainment of a goal). Scores for each subscale were obtained by summing the parent's score of each item for each subscale. The first three subscales were summed to comprise the Behavioral Regulation Index (BRI), while the next five were summed to comprise the Metacognitive Index (MI). Together the two indices form the Global Executive Composite (GEC; the child's overarching score of executive function). The BRIEF includes built-in checks for parent negativity and inconsistency in responses. The raw scores obtained from the eight subscales, two indices, and GEC are converted to standard scores based on age and gender norms provided in the BRIEF handbook (Gioia et al., 2000). In the present study, both raw and standardized scores were subjected to statistical analysis.

While the parent completed the three questionnaires in an area outside the testing lab, the child was welcomed into the lab with a “treasure map” and told that he/she could find a treasure by playing a few games (motor tasks) with the experimenter. The child participated in two tasks: grasp-to-construct and grasp-to-place. The tasks occurred in the same order for all participants. Tasks were video recorded with a JVC Everio HD camera positioned directly in front of the work-space, facing the seated participant and aligned with his/her midline. All children sat in chairs without armrests, and no directions were ever given regarding hand use.

#### Grasp-to-construct

**Figure 1:**
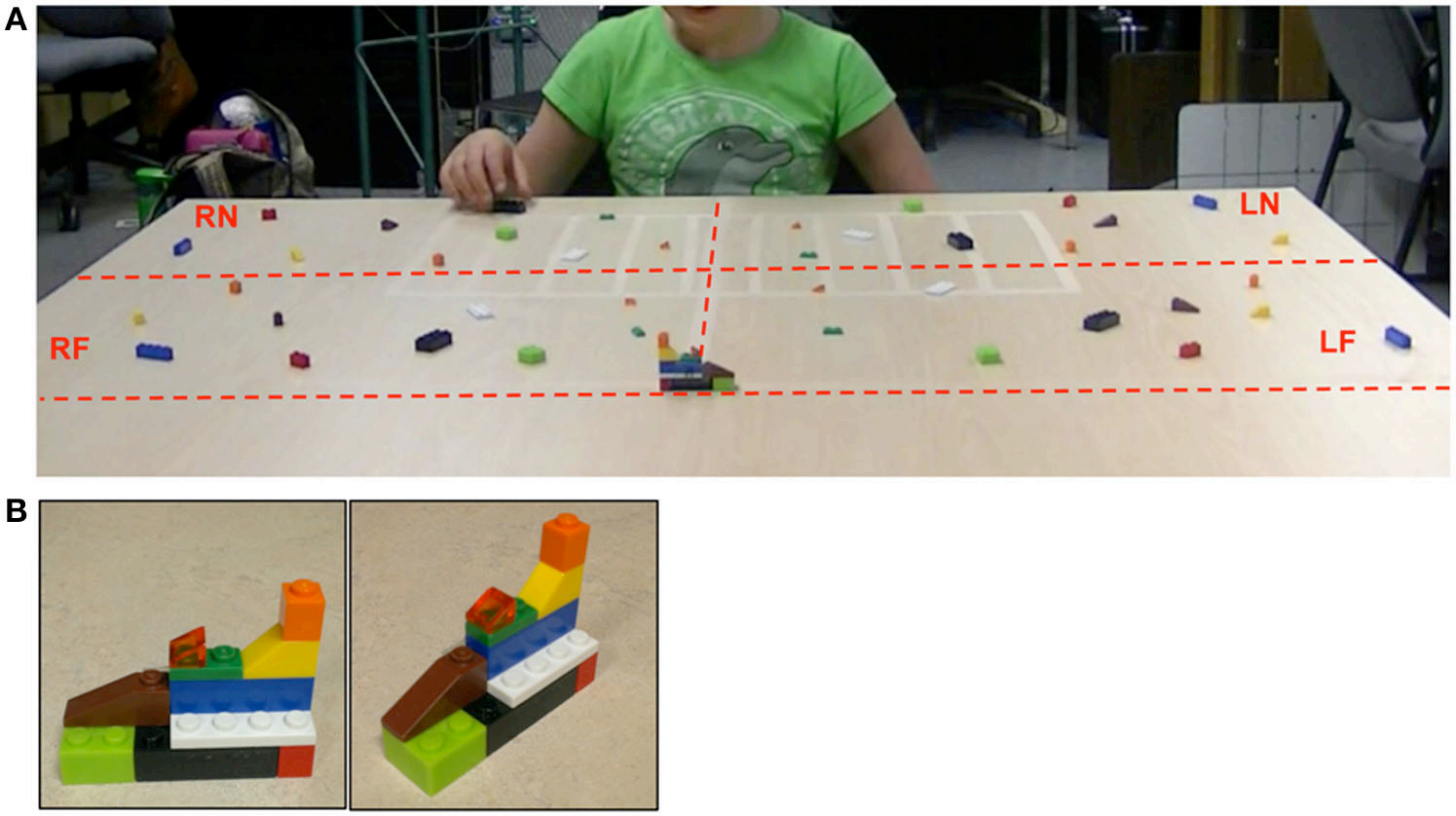
**(A)** The picture illustrates the workspace used by older children (9 and 10 years old) in the grasp-to-construct task. The table was notionally divided into four quadrants of equal dimensions (lines were not visible). Four identical sets of 10 pieces were placed on the tabletop—one set in each quadrant in near-mirror image placements. Within a set, pieces were unique in color and shape. The model to be replicated on each trial was placed at the far border of the workspace, aligned with the child's midline. **(B)** The figure demonstrates the first model older children were prompted to replicate in the grasp-to-construct task, from straight-on and side view angles. Each of the four models was composed of one piece set (contained in each quadrant on the table). Models were arranged such that they could be fully understood from a straight-on viewing angle, however, participants were allowed to pick up and rotate the model at any point during construction.

The child was asked to sit and face a table, with a workspace covered in Lego® blocks. The workspace was notionally divided into four quadrants of equal dimensions: left near (LN), left far (LF), right near (RN), and right far (RF). Each of the 4 quadrants contained the exact same set of pieces, which were unique in size, shape, and color within the set (see Figure 1A). In this task, the child was required to replicate four pre-made models. Each one was comprised of one set of pieces (the same set placed in each quadrant); thus, models contained the same pieces but in unique configurations. Within each age group, all children received the same four models, in the same order. The four sets of pieces on the table were placed in near-mirror image positions relative to one another, so that there was an equal opportunity to choose pieces from LN, LF, RN, or RF space when completing the models.

Individuals in the younger group (5–6 years old) sat at a table with a workspace 60 cm deep × 80 cm wide. These children encountered a total of 20 pieces on the tabletop; each of the four quadrants and four models contained the same set of five pieces. The older group (9–10 years old) sat at a table with a workspace 70 cm deep × 122 cm wide. These children encountered a total of 40 pieces (each quadrant and model contained the same set of 10 pieces).

Once seated, the experimenter explained to the child that the object of the “game” was to make a model that looked just like the experimenter's model. The experimenter gestured to a pre-made model, placed across from the child at the far end of the block array, aligned with the child's midline (see Figure 1A). Children in the older age group only were asked to complete the replica as quickly as possible. Children were allowed to pick up the original model at any point during the task, and manipulate it in any way to understand its configuration. However, models were designed to be fully understood from a straight-on viewing angle (see Figure 1B for an example). Once the first replica was complete, the experimenter removed the replica and replaced the first model with the next (in the same position). At the onset of the second trial, three sets of pieces were still available on the tabletop. After completion of all four replicas, all pieces on the table-top were used.

#### Grasp-to-place

**Figure 2:**
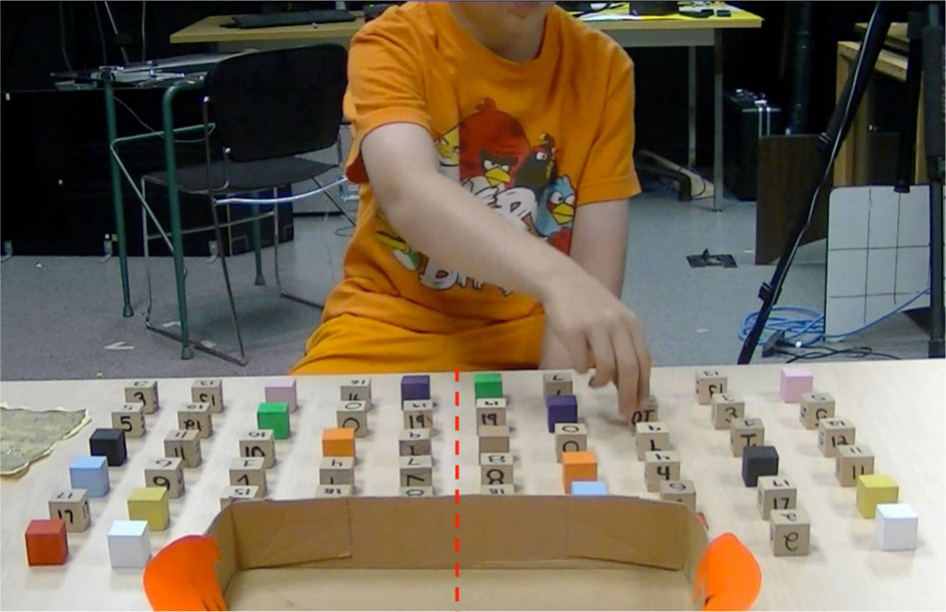
**An illustration of the workspace in the grasp-to-place task**. The table was notionally divided into left and right space; 2 identical sets of 20 numbered and 10 colored blocks were placed in left and right space in near-mirror image positions that remained consistent across participants. The experimenter called out a pseudo-random list of numbers and colors; after each, the child was to locate one correspondingly-labeled block as quickly as possible, and place it into the box at the far end of the array (the “monster's mouth”).

Immediately after the completion of the grasp-to-construct task, the child was seated at a table on which a total of 40 numbered and 20 colored blocks (2.54 cm^3^) were arranged in a rectangular array of six rows and 10 columns (see Figure 2). Blocks were placed approximately 6.35 cm apart, creating a grid approximately 33 cm deep × 61 cm wide. The grid was notionally divided into right and left space. One set of blocks (presented on one half of space) contained 20 blocks labeled with the numbers 0–19 and 10 blocks of different colors; blocks were placed in pseudo-random positions. In the other half of space, a replicate set of blocks was placed in a near-symmetric fashion. The placement of all 60 blocks was consistent across participants. At the far end of the array, a cardboard box 31.5 cm wide by 21.5 cm deep and decorated to look like a “monster's mouth” was placed.

The experimenter told the child that she was going to read a list of numbers and colors out loud. After each number or color, the child was to find and pick up one and only one corresponding block, and place it into the box. All participants were encouraged to be as fast as possible and no instruction as to what hand/space to use was given. Each number (0–19) and eight colors (28 requests total) were called out once in a pseudo-random order.

### Data processing and analysis

#### Brief

The BRIEF was scored according to scoring procedures outlined in the BRIEF handbook (Gioia et al., 2000). For each child, raw and standard scores were obtained for each component: the GEC, two indices (BRI and MI), and eight subscales.

#### Grasping tasks

All video recordings were analyzed offline.

#### Time-to-complete

Total latency to complete the four models in the grasp-to-construct task and the time required to place the numbered and colored bocks in the grasp-to-place task was recorded.

#### Hand use

Within each task, the hand used (left or right) for every grasp to a target item—a Lego® block or wooden block—was scored. The total number of grasps was calculated to determine the percentage of right hand use [(number of grasps with right hand/total number of grasps) ×100] for each individual on each task.

#### Space use in the grasp-to-construct task

In a previous study from Gonzalez' lab using the grasp-to-construct task with adults (de Bruin et al., 2014), differential use of space for grasping (left vs. right and near vs. far) was shown. Right-handed participants grasp from right-near space earlier than anywhere else. We explored the possibility that adult-typical patterns of space use in children would be correlated with better EF. Space use in the grasp-to-construct task was investigated by assigning a number to each grasp based on the order in which the grasp occurred (the first grasp received a 1, the second a 2, the third a 3, and so forth). At task completion, each quadrant had five grasp values assigned to it for the younger group. For example, if the first five grasps made by a participant occurred in the right near quadrant, the values 1, 2, 3, 4, and 5 would be assigned to that quadrant. Within each quadrant, values were then summed to produce four quadrant sums and two hemi-space sums (L and R). The lowest possible quadrant sum for the younger group was 15 (1, 2, 3, 4, and 5), and the highest possible sum was 90 (16, 17, 18, 19, 20). In the older group, 10 pieces were placed in each quadrant, raising the minimum quadrant sum to 55 and the maximum to 355. Each quadrant and hemi-space sum was then divided by the table sum (210 in the younger group, 820 in the older group), to obtain quadrant and hemi-space percentages. The lower the percentage for a given space, the earlier in the task that space was attended to and exhausted of pieces (de Bruin et al., 2014).

All data were analyzed using SPSS Statistics 19.0 for Mac (SPSS Inc., Chicago, IL, USA). Statistical significance was set at α = 0.05. Correlation (Pearson's *r*) and regression analyses (linear) between scores from the BRIEF and scores from the grasping tasks were computed. In addition, means and standard errors for the time-to-complete and hand use for grasping are reported below. The results were analyzed for overall effects (both age groups together) and then inspected separately for each age group. Only significant results are reported.

## Results

No statistically significant differences were found with respect to sex in either age group or in any of the measurements, therefore the data was collapsed across this variable.

### Descriptive statistics

In the BRIEF, lower scores are associated with better EF. Table 1 shows the results for children in the two age groups for each of the components of the BRIEF. In the grasp-to-construct task the younger group spent on average 141.42 ± 10.41 (SEM) s completing the task whereas the older group spent on average 191.95 ± 8.4 s. The older group required more time to complete the task because they were presented with 40 Lego® blocks instead of the 20 blocks the younger group worked with. In the grasp-to-place task the younger group spent on average 250.73 ± 15.41 s completing the task whereas the older group spent on average 114.95 ± 5.0 s. In this case, both groups were presented with the same number of wooden blocks.

**Table 1:** **Mean standard scores and standard deviations on the eight subscales, two indices, and General Executive Composite of the BRIEF, for all participants and the two separate age groups**.

**BRIEF component**	**All ages**	**Younger**	**Older**
General Executive Composite (GEC)	53.65 (±10.56)	56.05 (±10.74)	51.48 (±10.16)
Behavior Regulation Index (BRI)	54.35 (±10.4)	58.11 (±10.47)	50.95 (±9.32)
Inhibit	53.05 (±12.11)	57.68 (±14.03)	48.86 (±8.37)
Shift	54.9 (±12.21)	57.21 (±12.72)	52.81 (±11.63)
Emotional control	53.75 (±10.27)	56.63 (±8.86)	51.14 (±10.96)
Metacognitive Index (MI)	52.7 (±10.69)	53.58 (±10.53)	51.9 (±11.03)
Initiate	52.35 (±10.13)	52.26 (±10.94)	52.43 (±9.6)
Working memory	54.55 (±11.07)	56.79 (±10.97)	52.52 (±11.03)
Plan/Organize	51.1 (±9.02)	51.56 (±7.18)	50.71 (±10.5)
Organization of materials	53.55 (±10.1)	54.79 (±9.54)	52.43 (±10.7)
Monitor	51.45 (±11.56)	53.63 (±11.96)	49.48 (±11.09)

In the grasping tasks, both groups of children displayed a right hand preference. In the grasp-to-construct task, percent right hand use in the younger children was 59.82 ± 12.42 and the older children 68.11 ± 14.23. In the grasp-to-place task these values were 74.47 ± 23.74 and 85.54 ± 17.84, respectively.

Children of both ages displayed a preference for attending first to right space and specifically to right-near space. In the younger group, percent right hemispace use was 44.89 ± 10.28 and the older group was 40.44 ± 8.29. For the right near quadrant, sum averages were 15.74 ± 5.95 and 17.65 ± 6.25, respectively.

### Correlation analyses using BRIEF standard scores

Our main hypothesis was that measures of motor performance would correlate with executive function. The dependent variables in both grasping tasks were the time that participants took to complete each task and the hand used to pick up the blocks. We hypothesized that faster times in completing the tasks, particularly in the grasp-to-construct, would correlate with better EF. We had no particular prediction regarding hand use and its possible relationship with EF. In addition, space use was documented during the grasp-to-construct task to explore the possibility that children exhibiting adult-typical space use (right-handed participants attend to right-near space first) in the grasp-to-construct task would have better EF scores.

No significant correlations were found for the standard scores of the BRIEF and the time to complete either grasping task.

As mentioned previously, lower scores on the BRIEF indicate better EF. Therefore, a negative correlation between right hand use and EF would indicate that the more the right hand is used for grasping the better the EF score.

Overall (age groups combined), there was a significant negative correlation between **hand use** in the **grasp-to-construct task** and the standard score on the Inhibit subscale of the BRIEF [*r*_(40)_ = −0.39; *p* < 0.02]. A closer look at this correlation revealed the significant effect was mostly driven by the younger children [*r*_(19)_ = −0.52; *p* < 0.03]. In addition, when looking at this young group a significant correlation was also found between right hand use and the score on the Monitor subscale [*r*_(19)_ = −0.62; *p* < 0.01]. Furthermore, trends were noted for Emotional Control [*r*_(19)_ = −0.41, *p* = 0.09], BRI [*r*_(19) = −0.45_, *p* = 0.05], and GEC [*r*_(19)_ = −0.41, *p* = 0.08]. No other significant correlations were found for any of the remaining subscales or age groups.

**Figure 3:**
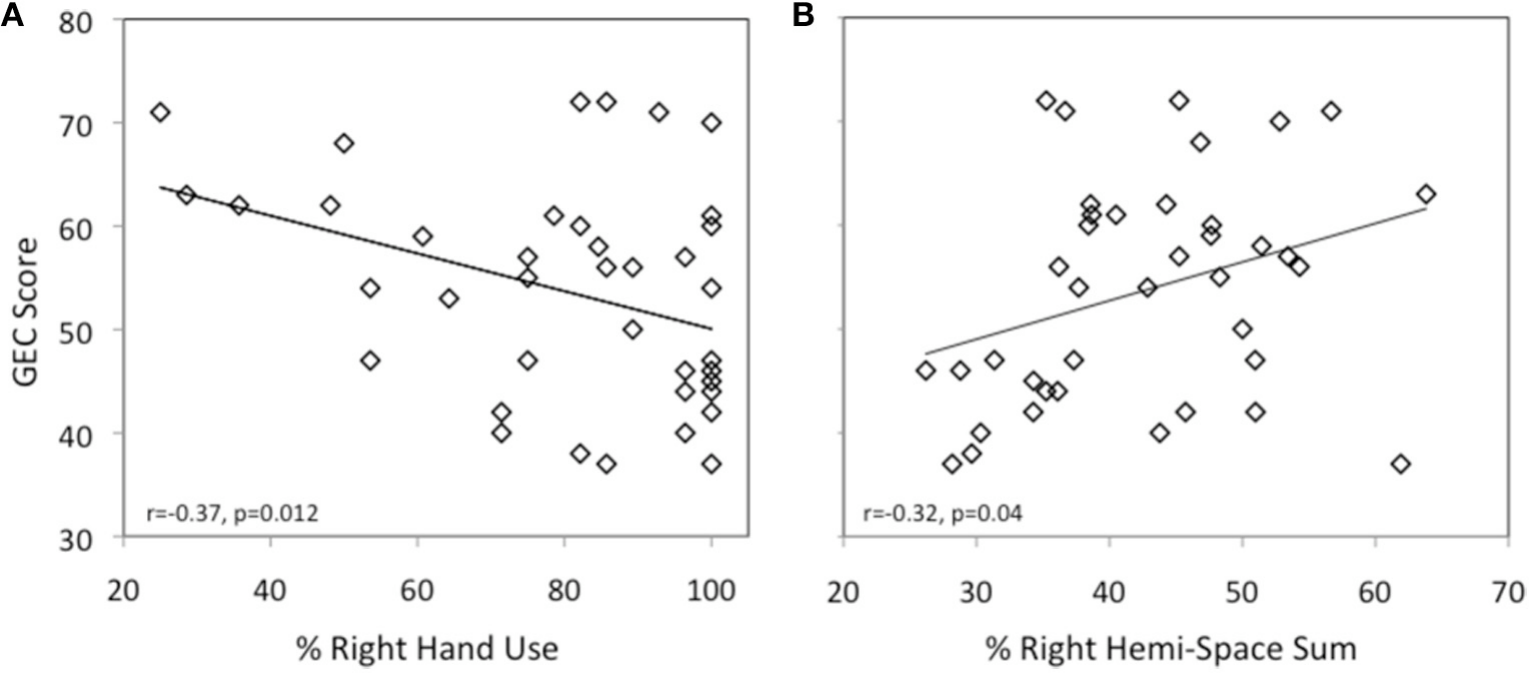
**(A)** The graph depicts the relationship between percent right hand use in the grasp-to-place task and the standard score obtained on the General Executive Composite of the BRIEF for all children (younger and older). A significant negative correlation was observed (*r* = −0.368, *p* = 0.019), indicating that the more the right hand was used for grasping, the lower (better) the overarching EF score. **(B)** The graph depicts the relationship between percent right hemi-space sum in the grasp-to-place task and the standard score obtained on the General Executive Composite of the BRIEF for all children (younger and older). A smaller percent sum indicates earlier attendance to the right space. A significant positive correlation was observed (*r* = 0.327, *p* = 0.042), demonstrating that the earlier the right space was attended to, the lower (better) the overarching EF score.

For the **grasp-to-place task** overall, there was a significant negative correlation between **hand use** and the standard GEC score [*r*_(40)_ = −0.37; *p* < 0.02; see Figure 3A]. Furthermore, the correlation was maintained across the two indices; BRI [*r*_(40) = −0.33_; *p* < 0.05] and MI [*r*_(40)_ = −0.35; *p* < 0.05]. Closer examination revealed significant correlations for Inhibit [*r*_(40)_ = −0.43; *p* < 0.01], Working Memory [*r*_(40)_ = −0.32; *p* < 0.05], Plan [*r*_(40) = −0.35_; *p* < 0.05], and Monitor [*r*_(40)_ = −0.42; *p* < 0.01]. When separated by age, the correlation held for Monitor [*r*_(19)_ = −0.54; *p* < 0.02] and a trend for Plan was observed [*r*_(19)_ = −0.40; *p* = 0.09] in the younger group. For the older group, a trend was observed for Inhibit [*r*_(21)_ = −0.40; *p* = 0.07].

As previously stated, we explored the possibility that children exhibiting adult-typical **space use** in the grasp-to-construct task would have better EF scores. Lower scores on any space sum (%) are indicative of children attending to that space earlier (see Materials and Methods). A positive correlation between space sum and the scores of the BRIEF indicate that the earlier a child attends to that space, the better the EF. Results showed that the earlier the **right hemispace** was attended to, the better the EF score. Overall there was a significant positive correlation between **right hemispace sum (%)** and the standard GEC score [*r*_(40)_ = 0.33; *p* < 0.05] (see Figure 3B). Closer examination revealed a significant positive correlation for Plan [*r*_(40)_ = 0.36; *p* < 0.05]. In addition, trends were observed for the two indices; BRI [*r*_(40)_ = 0.31; *p* = 0.057] and MI [*r*_(40)_ = 0.30; *p* = 0.068], and the subscales Inhibit [*r*_(40)_ = 0.31; *p* = 0.058], Working Memory [*r*_(40)_ = 0.28; *p* = 0.083], and Monitor [*r*_(40)_ = 0.31; *p* = 0.054]. These effects were mostly driven by the older group. For this group there was a significant positive correlation between **right hemispace sum (%)** and the standard GEC score [*r*_(21)_ = 0.59; *p* < 0.01]. Significant positive correlations were also found for MI [*r*_(21)_ = 0.61; *p* < 0.005], and the subscales Initiate [*r*_(21)_ = 0.43; *p* = 0.05], Working Memory [*r*_(21)_ = 0.61; *p* < 0.005], Plan [*r*_(21)_ = 0.65; *p* < 0.005], and Organization of Materials [*r*_(21)_ = 0.44; *p* < 0.05]. Trends were observed for BRI [*r*_(21)_ = 0.39; *p* = 0.08], and the subscales Inhibit [*r*_(21)_ = 0.39; *p* = 0.079], and Monitor [*r*_21)_ = 0.43; *p* = 0.05]. Again, the earlier the right space was attended to, the better the EF score. We further investigated the hemi-space effect in the older group by looking at the **right near quadrant space use (%)** and found that the earlier the right near quadrant was attended to, the better the EF score. Consistent with our hypothesis, significant positive correlations between right-near space sum (%) were found for the standard GEC score [*r*_(21)_ = 0.57; *p* < 0.01], MI [*r*_(21)_ = 0.56; *p* < 0.005], Inhibit [*r*_(21)_ = 0.46; *p* < 0.05], Initiate [*r*_(21)_ = 0.51; *p* < 0.02], Working Memory [*r*_(21)_ = 0.63; *p* < 0.005], Plan [*r*_(21)_ = 0.57; *p* < 0.01], and Organization of Materials [*r*_(21)_ = 0.53; *p* < 0.02].

### Correlation analyses using BRIEF raw scores

Because it is known that EF improves with developmental age (for a review see Best and Miller, 2010) we wondered whether right hand use increases as well-with developmental age and if therefore our results could be explained on the basis of age alone. In other words, we investigated whether the relationship between hand use and EF score is an epiphenomenon of hand use changing with age (i.e., whether children get more right-handed as they age). We found no significant correlation between chronological age (days) and right hand use in either grasping task: grasp-to-construct [*r*_(40)_ = 0.39; *p* > 0.05] or grasp-to-place [*r*_(40)_ = 0.24; *p* > 0.1].

Given that the BRIEF standardizes raw scores to normative data for age, we explored possible correlations between chronological age (days) and raw BRIEF scores. We found a significant negative correlation between chronological age and the BRI [*r*_(40)_ = −0.34; *p* < 0.05] as well as the Inhibit [*r*_(40)_ = −0.41; *p* < 0.01] subscale of the BRIEF; the older the child the better their EF score.

Unexpectedly, we found more significant correlations between the BRIEF raw scores and hand use, than the BRIEF raw scores and age. Overall (both ages combined), there was a significant correlation between right hand use in the grasp-to-construct task and the raw scores on the Inhibit subscale [*r*_(40)_ = −0.44; *p* < 0.005]. In the grasp-to-place task, right hand use correlated raw scores on the GEC [*r*_(40)_ = −0.36; *p* < 0.05], MI [*r*_(40)_ = −0.32; *p* < 0.05], BRI [*r*_(40)_ = −0.34; *p* < 0.05], Monitor [*r*_(40)_ = −0.37; *p* < 0.02], and Inhibit [*r*_(40)_ = −0.47; *p* < 0.002]. Further analysis revealed that the observed correlations were mostly driven by the younger group. Within this group significant correlations were found for Inhibit [*r*_(19) = −0.577_, *p* = 0.01], BRI [*r*_(19)_ = −0.498, *p* = 0.03], Monitor [*r*_(19) = −0.614_, *p* = 0.007], borderline GEC [*r*_(19) = −0.444_, *p* = 0.057], and Emotional Control [*r*_(19) = −0.409_, *p* = 0.082].

### Regression analyses

To explore the contributions that age, hand-use, and space-use had on EF we conducted several linear regression analyses. Given that the grasp-to-place task yielded more and stronger correlations of right-hand use with EF, we used this measure in the hand use regression analyses. For space-use, right near quadrant sum was used in the computation. For simplicity we focused on the GEC as the dependent measure. The model accounted for 15.7% of the variance, and it was significant [*F*_(3, 39)_ = 3.4; *p* < 0.05]. An examination of the coefficients showed that right hand use and right-near space use were significant predictors of EF (see Table 2). Interestingly, age was not a predictor of EF. To explore the possibility of a mutually predictive relationship, we computed a second regression analysis with right-hand use as the dependent measure and chronological age, GEC, and space use as independent measures. The model accounted for 12.0% of the variance and it was significant [*F*_(3, 39)_ = 2.8; *p* = 0.05]. Examination of the coefficients showed that GEC was a significant predictor of right-hand use (see Table 2). Neither chronological age nor space use predicted right-hand use. A final regression analysis was conducted to investigate if chronological age, hand-use and GEC would be predictors of space use. The model accounted for 64.3% of the variance and significance was noted [*F*_(3, 39)_ = 24.4; *p* < 0.0001]. The coefficients revealed that chronological age was a powerful predictor of right-near space use (see Table 2). GEC was also a predictor of space use but hand use was not.

**Table 2:** **Results of the regression analyses. Note the relationship between hand and space use during the grasping tasks and EF**.

**Dependent measure**		**Coefficients**							
		**Unstandardized coefficients**		**Standardized coefficients**				**Correlations**	
		***B***	**Std. error**	**Beta**	***t***	**Sig**.	**Zero-order**	**Partial**	**Part**
**GEC**									
	Chrono-age	−0.013	0.008	−0.39	−1.61	0.11	−0.08	−0.25	−0.23
	RH-use	−41.97	17.41	−0.36	−2.40	**0.02**	−0.36	−0.37	−0.35
	RN-space use	0.180	0.088	0.49	2.05	**0.04**	0.11	0.32	0.30
**RIGHT-HAND USE**									
	Chrono_age	0.00	0.00	0.04	0.17	0.86	0.23	0.03	0.02
	RN-space use	0.001	0.001	0.20	0.77	0.44	0.19	0.12	0.11
	GEC	−0.003	0.001	−0.38	−2.40	**0.02**	−0.36	−0.37	−0.36
**RIGHT-NEAR SPACE USE**									
	Chrono_age	0.07	0.009	0.79	8.05	**0.000**	0.79	0.80	0.77
	RH-use	25.97	33.52	0.08	0.77	0.444	0.19	0.12	0.07
	GEC	0.58	0.28	0.21	2.05	**0.047**	0.11	0.32	0.19
**CHRONOLOGICAL AGE**									
	RN-space use	8.83	1.09	0.81	8.05	**0.000**	0.79	0.80	0.77
	RH-use	66.07	372.1	0.01	0.17	0.86	0.23	0.03	0.01
	GEC	−5.14	3.19	−0.17	−1.61	0.11	−0.08	−0.25	−0.15

The bolded values represent the significance.

## Discussion

The purpose of the present study was to investigate the possible relationship between motor performance and EF. To do this we asked children of two different ages to complete two grasping tasks while their parents filled out a questionnaire detailing their child's EF. For the grasping tasks, children reached for and grasped Lego® blocks in order to construct different models, or grasped wooden blocks to place in a box. Three aspects of their performance were assessed: the time it took them to complete each task, their preference for hand use, and their preference for space use. The results showed no relationship between EF and their performance as measured by time. In other words, how quickly a child completed the tasks bore no relationship to their scores on the BRIEF. However, the results demonstrated a robust relationship between the scores on the BRIEF and the child's preference to use their right hand and the right space for grasping. Remarkably, right hand use and right space use were predictors of EF, and EF was a reliable predictor of right hand use. These unexpected findings suggest that a more lateralized brain supports enhanced EF.

Studies have shown overlapping neural networks that support motor and EF including the frontal lobe, the cerebellum, and the basal ganglia (Schmahmann and Pandya, 2008; Abe and Hanakawa, 2009; Pangelinan et al., 2011; for a review see Diamond, 2000). At the behavioral level, numerous studies have presented evidence of motor deficits accompanying cognitive deficits (e.g., Eliason, 1986; Eliasson et al., 2004; Racine et al., 2008; Fuentes et al., 2009). Children with developmental coordination disorder for example, present with a host of gross and fine motor skill deficits. Up to 50% of these children may suffer from executive dysfunction (Willcutt and Pennington, 2000; Sugden et al., 2008) that in some cases lasts into the adult life (Kirby et al., 2008). Furthermore, fine motor skills have been used as the primary indicator of the need for intervention in kindergarten children (Roth et al., 1993). In normally developing children, studies have also reported a relationship between EF and motor performance (e.g., Roebers and Kauer, 2009; Davis et al., 2011; also Piek et al., 2008). Cameron et al. (2012) tested children in several gross and fine motor tasks and discovered that children that were better at a design copy task requiring fine motor control (copy pictures of different geometrical shapes using paper and pencil) not only performed better on tests of EF, but they also attained higher kindergarten achievement. Recently, Carlson reported that children starting kindergarten with better fine motor skill showed enhanced learning in both math and reading (Carlson et al., 2013). Based on these previous examples we hypothesized that performance measures such as time to complete the grasping task might predict EF. This was not the case. In reviewing the video footage it was obvious that individual differences contributed to noise in this measure. For example, some children were more familiar with assembling Lego, some were very verbally interactive with the experimenter, and yet others seemed shy or introverted. These factors likely undermined the effectiveness of time as a measure of performance.

Although time to complete the grasping tasks did not correlate with any measures of the BRIEF, we found that the strength of right hand and space preference was intimately related with EF. Results from the present study suggest two potential and non-mutually exclusive scenarios: (1) the possibility that EF enjoys privileged support from the left hemisphere; and/or (2) that the greater the lateralization of function (either to the left or right hemisphere), the better the behavioral output. With respect to the first scenario, there is reasonable, albeit not explicit, evidence of increased involvement of the left hemisphere in EF. In a recent study, a large sample of brain-injured adults was subjected to neuropsychological testing and brain imaging analysis (Barbey et al., 2012). Both hemispheres were scanned for evidence of injury. Interestingly, the results showed that high-level cognitive performance (intelligence and EF) was compromised in patients with left hemisphere damage only. In a similar study of brain-damaged patients, measures of general intelligence (some of which overlap with EF) were correlated with a left lateralized fronto-parietal network (Glascher et al., 2010). Furthermore, this study identified a sector in the left anterior frontal lobe (BA 10) that was uniquely related to general intelligence. Curiously, BA 10 has also been implicated in the planning of movement (Momennejad and Haynes, 2013) and specifically a relationship has been found between better motor imagery and activation of the left “prefrontal executive” area BA10 (van der Meulen et al., 2012). In light of this evidence, it is perhaps not surprising that our participants that showed more left hemisphere lateralized biases for hand and space use also showed higher EF scores. In other words, our results provide strong evidence of left hemisphere specialization for EF.

The second possibility is that a greater degree of functional lateralization supports better motor and cognitive performance. Indeed, there is evidence to support this notion. In a study by Crow et al. (1998) 12,770 children were assessed for hand skill and cognitive control. For the hand skill task, children were given 1 min to put a check mark in as many squares as possible on a printed sheet of paper. In two separate trials participants used their right or their left hands. The authors found that the most substantial deficits in the cognitive tasks (verbal, non-verbal, reading comprehension, and mathematical ability) corresponded to those children that were closer to the point of equal hand skill, exhibiting what they called “hemispheric indecision” (Crow et al., 1998). The authors suggest that failure to establish hemispheric dominance unequivocally is problematic and that lack of dominance by age 11 results in global delays in cognitive development. Supporting this finding, a more recent study showed that children with consistent hand use and superior skill of the preferred hand obtained better scores in reading and mathematics (Cheyne et al., 2010). Other studies, however, have failed to find a relationship between lateralized hand use and cognitive abilities (Mayringer and Wimmer, 2002). Crow et al. (1998) however, suggested that this might be attributed to a failure in appreciating handedness as a continuum rather than an absolute. Our results support this view because rather than considering children as right-handed or left-handed, their hand preference was evaluated by hand use in a natural (unconstrained as to what hand or grip to use) grasping task. In our experiment, all children self-reported as right-handed, yet many of them failed to show a clear right hand preference for grasping. Overall, these children's BRIEF scores indicated more problems with executive function. In other words, our grasping tasks produced a continuum of right hand use rather than an absolute preference that correlated and more importantly, predicted EF. It remains to be shown if left-handed children that display a very strong left hand preference (thus strong right hemisphere lateralization) also enjoy enhanced EF. Regardless of handedness, if the degree of lateralization supports better motor and cognitive performance, then we would predict that very strongly left-handed individuals would show similar advantages to those with a strong right hand preference.

Developmental research has provided evidence that by birth, both anatomical and functional lateralization are features of the human brain (for a recent review see Hervé et al., 2013). Furthermore, studies have shown that compared to other brain circuits, regions subserving motor control are established and refined earlier (Lin et al., 2008; Dubois et al., 2009; Ratnarajah et al., 2013). Ratnarajah et al. used DTI to determine the pattern of structural connectivity asymmetry in 124 normal neonates. Their results showed that the left hemisphere exhibits greater structural efficiency than does the right hemisphere, and they conclude that this early specialized connectivity supports lateralized functional need, particularly in the motor domain. This evidence suggests that anatomical asymmetries exist at birth and functional lateralization continues to mature during childhood (Hervé et al., 2013). Our results are in line with these findings. Children in the older age group displayed greater preference for using their right hand during grasping as well as lower scores on the BRIEF, which indicates better EF. Although speculative, it is possible that greater structural efficiency in the left hemisphere contributes to stronger right hand preference and EF. Clearly this relationship deserves further consideration. Our results suggest the interesting possibility of utilizing measures of motor lateralization for predicting deviations from normal developmental trajectories, specifically for EF. This suggestion would be supported by studies showing the power of using motor skill as a predictor of later cognitive abilities. For example Johnson et al. (1995) showed that fine motor tasks predict kindergarten readiness and other have found correlations between fine motor skills and reading and mathematical achievement (Wolff et al., 1985; Luo et al., 2007). To our knowledge no study has introduced measures of hand and space lateralization as a tool to assess cognitive function, let alone as a means to enhance these processes. We speculate that those studies showing that better fine motor skill correlate with better cognitive abilities might be in part related to the strength of hand preference (i.e., lateralization). It is well-known that proficiency in a manual activity is related to the amount of practice during the learning period (e.g., Jabusch et al., 2009). Furthermore, it has been shown that training-induced brain plasticity after motor sequence learning persists for months (Karni, 1995). We propose that working on hand skills that promote lateralization might be an effective method to enhance EF.

A strength of the current study was the degree to which hand and space use correlated and further predicted the GEC of the BRIEF. Furthermore, both indices and many subscales of the BRIEF correlated with hand and space use. The only subscale that never correlated with any of the grasping measures was *shift*. This is not surprising, as we believe our tasks did not require the child to shift problem-solving strategies to be successful. However, it is important to bear in mind that shift contributes to both the Behavior Regulation Index (BRI) and ultimately the GEC. Both of these measures repeatedly correlated with grasping behavior. The subscales of the BRIEF that appeared most often as significantly correlated with our grasping measures were *inhibit, plan*, and *working memory*. As defined by Gioia and Isquith (2004), “inhibit is the ability to resist or delay an impulse, to appropriately stop one's own activity at the proper time, or both; plan involves anticipating future events, setting goals and developing appropriate steps ahead of time to carry out an associated task or action; working memory is the process of holding information in mind for the purpose of completing a related task.” Both grasping tasks demand recruitment of these three components for successful completion. For example, in the grasp-to-construct the child must: (1) resist the impulse of grabbing all the pieces at once, and/or assembling a structure of their own design (*inhibit*), (2) develop the appropriate steps ahead of time to reproduce the sample model (*plan*), and (3) keep in memory the goal of the task (*working memory*). Similarly, in the grasp-to-place task the child must wait, listen to, and follow the instruction as to which blocks to grasp (inhibit, planning, working memory). In both cases a motor plan must be created and executed in order to grasp the blocks. We believe these tasks tested the fundamental essence of the *inhibit, plan*, and *working memory* subscales. Our results align with a trend in the literature which has shown *inhibit*, *plan*, and *working memory* as a reliable measures of EF (Moriguchi and Hiraki, 2013; for reviews see: Jurado and Rosselli, 2007; Best and Miller, 2010).

The results from the regression analyses highlight the interconnectedness of EF and lateralization for hand and space use. To find out which variables were useful as predictors of others, chronological age, right-hand use, right-near space use, and GEC were each used separately as dependent measures. Notably, we found that both hand and space use are predictors of EF. In turn, EF is a predictor of right hand use and space use. In other words, the more children used their right hand or the right near space for grasping, the better their EF scores and vice versa. This is a remarkable finding that could have implications for intervention. There is emerging evidence that motor activity such as aerobic exercise (Hillman et al., 2008; Chaddock et al., 2011), bimanual basketball dribbling (Davis et al., 2011) and handwriting (Rosenblum, 2013) improves aspects of executive function. What remains to be shown is whether short-term motor interventions that promote the use of the right hand during skill grasping have a beneficial effect on EF. The regression analyses also showed a reciprocal relationship between chronological age and right-near space use. The older the child, the more likely they are to grasp in right near space first and vice versa. This result is consistent with our hypothesis that as children age their use of space resembles the adult pattern, that is, right-handed adults prefer to grasp in right-near space followed by equal use of left-near and right-far space (de Bruin et al., 2014). The results suggest that there is a maturation time-line for space use. In light of the current results, these issues warrant further investigation.

A limitation of this study was the exclusive use of the BRIEF as our measure of EF. Clearly additional in-house tests of EF would both inform and complement the assessment of these processes. Future investigations aimed at a more comprehensive assessment of EF might further substantiate the current findings.

In conclusion, the results from the present investigation suggest finer measures that afford an examination of hand and space use preference for grasping should be included to complement existing strategies for early detection of developmental delays, particularly if EF truly predicts school achievement and life success.

### Conflict of interest statement

The authors declare that the research was conducted in the absence of any commercial or financial relationships that could be construed as a potential conflict of interest.
